# Systematic sequencing of imported cases leads to detection of SARS-CoV-2 B.1.1.529 (Omicron) variant in central Viet Nam

**DOI:** 10.5365/wpsar.2022.13.4.977

**Published:** 2022-11-23

**Authors:** Do Thai Hung, Nguyen Bao Trieu, Do Thi Thu Thuy, Allison Olmsted, Trinh Hoang Long, Nguyen Duc Duy, Huynh Kim Mai, Bui Thi Thu Hien, Nguyen Van Van, Tran Van Kiem, Vo Thi Thuy Trang, Nguyen Truong Duy, Ton That Thanh, Huynh Van Dong, Philip Gould, Matthew Moore

**Affiliations:** a Pasteur Institute in Nha Trang, Nha Trang, Viet Nam.; b U.S. Centers for Disease Control and Prevention, Hanoi, Viet Nam.; c Quang Nam Department of Health, Quang Nam, Viet Nam.; d Quang Nam Center for Disease Control, Quang Nam, Viet Nam.; e Da Nang Center for Disease Control, Da Nang, Viet Nam.; f Khanh Hoa Center for Disease Control, Viet Nam.

## Abstract

As authorities braced for the arrival of the Omicron variant of severe acute respiratory syndrome coronavirus 2 (SARS-CoV-2), infrastructure investments and government directives prompted action in central Viet Nam to establish capacity for genomic surveillance sequencing. From 17 November 2021 to 7 January 2022, the Pasteur Institute in Nha Trang sequenced 162 specimens from 98 150 confirmed SARS-CoV-2 cases in the region collected from 8 November to 31 December 2021. Of these, all 127 domestic cases were identified as the B.1.617.2 (Delta) variant, whereas 92% (32/35) of imported cases were identified as the B.1.1.529 (Omicron) variant, all among international flight passengers. Patients were successfully isolated, enabling health-care workers to prepare for additional cases. Most (78%) of the 32 Omicron cases were fully vaccinated, suggesting continued importance of public health and social measures to control the spread of new variants.

Genomic surveillance has become a critical tool for monitoring variants of severe acute respiratory syndrome coronavirus 2 (SARS-CoV-2), the virus that causes coronavirus disease (COVID-19). (*1*) Recognizing this, capacity building for genomic surveillance towards a National Sequencing Network has become a principal goal in Viet Nam. The aim of the network is to standardize sequencing to empower the Ministry of Health (MoH) to detect and respond to emerging public health threats, determine outbreak etiologies and enhance overall surveillance capacities in the country. The Pasteur Institute of Nha Trang (PI Nha Trang) is the agency within the MoH responsible for public health surveillance and response in 11 central coastal provinces in Viet Nam, including key population centres such as Da Nang as well as major tourist destinations such as Hoi An and Nha Trang. In May 2021, motivated by the desire to participate in the development of the National Sequencing Network, PI Nha Trang acquired its first next-generation sequencer and developed a protocol to identify and monitor the relative prevalence of SARS-CoV-2 variants among COVID-19 cases identified in the central region.

In early 2020, all passengers arriving on inbound flights to Viet Nam underwent testing using real-time reverse transcription polymerase chain reaction (RT–PCR) (requirement expired 15 May 2022) and mandatory 14-day quarantine in centralized facilities (requirement expired 1 January 2022). (*2*) These provisions allowed for quick detection and isolation of cases, as well as targeted genomic surveillance for imported cases.

The B.1.1.529 (Omicron) variant of SARS-CoV-2 was first reported to the World Health Organization by South Africa and was designated a variant of concern on 26 November 2021. (*3*) It has since been closely monitored by countries worldwide. Through heightened genomic surveillance measures, the first imported case of the Omicron variant in Viet Nam was detected on  19 December 2021 in a traveller entering the northern part of the country. This brief report characterizes 32 of Viet Nam’s first imported Omicron cases in the central region and highlights the importance of public health and social measures to detect and contain the spread of new variants.

## Methods

Specimens were collected from domestic and imported laboratory-confirmed cases of COVID-19 from 8 November to 31 December 2021. Not all COVID-19 cases were sequenced; each province was required to submit 2–3 specimens per week, while areas with international airports (Khanh Hoa and Da Nang provinces), border controls (Quang Binh and Quang Tri provinces) or a high case burden submitted 4–6 specimens per week. The selection of samples for sequencing was prioritized to imported cases or special cases (e.g. index cases of large clusters, patients infected after two vaccine doses, reinfected, or with severe symptoms or death).

Sequencing was performed on specimens with a cycle threshold value of £28 and volume of at least 800 µL, and with accompanying epidemiological data such as patient characteristics and clinical presentation. Specimens were stored at 2–8 °C for £72 hours from the time of collection. PI Nha Trang has the capacity to sequence 24–46 specimens per week using a MiSeq platform (Illumina, San Diego, CA, United States of America). The Dragen Covid Lineage software (Illumina) was used for genomic analysis and phylogenetic trees were generated using Nextclade (https://clades.nextstrain.org/). Outputs included quality metrics, lineage determination, amino acid substitutions for novel strain detection, and Fasta format files for epidemiological analysis using accompanying epidemiological data.

Sequences were posted to the GISAID Initiative database as per the protocol developed jointly by PI Nha Trang and the US. Centers for Disease Control and Prevention in Viet Nam (unpublished). To improve understanding of the effects of systematic testing and quarantine, sequence data for community cases were reported from November 2021 to February 2022.

## Results

There were 98 150 confirmed cases of COVID-19 detected in central Viet Nam (97 985 domestic and 165 imported) from 1 November to 31 December 2021. Among all cases, 162 patient specimens collected between 8 November and 31 December 2021 were sequenced. The majority (78%; 127/162) were domestic cases while 22% (35/162) were imported cases. All domestic cases were identified as the B.1.617.2 (Delta) variant while 92% (32/35) of imported cases were the Omicron variant. Phylogenetic relationships among sequenced cases demonstrate close relationships with sequences from multiple continents (**Supplementary Fig. 1**). No community cases of Omicron were detected in the 11 provinces in central Viet Nam from November 2021 to early February 2022.

Descriptive epidemiology revealed that 66% (21/32) of Omicron cases were under 40 years old, 53% (25/32) were fully vaccinated and 25% (8/32) had received booster doses (**Table 1**). Most of the cases (24/32; 75%) were asymptomatic at detection. None were hospitalized or died. A total of 13 individuals arrived on direct flights from the United States of America to Viet Nam and 13 additional passengers departed from various countries with stopover points in the Republic of Korea, where layover time ranged from 4 to 30 hours.

**Table 1:** Characteristics of confirmed B.1.1.529 (Omicron) SARS-CoV-2 cases (*n* = 32), central Viet Nam, 8 November–31 December 2021

Characteristic	No.	%
**Age group (years)**		
0–19	3	9
20–29	12	38
30–39	6	19
40–64	11	34
^3^65	0	0
**Sex**		
Male	12	38
Female	20	63
**COVID-19 vaccination status^a^**		
Fully vaccinated	17	53
Fully vaccinated plus booster dose	8	25
Unvaccinated (ineligible)	1	3
Unknown	6	19
**Symptom profile^b^**		
Asymptomatic	16	50
Symptomatic	8	25
Unknown	8	25
**Initial signs or symptoms^c^**		
Cough	4	13
Sore throat	3	9
Fever	2	6
Congestion or runny nose	1	3
Headache	1	3
Nausea or vomiting	1	3
**Outcome**		
Hospitalization	0	0
Death	0	0
**Flight origin**		
Republic of Korea^d^	16	50
United States of America	13	41
Malaysia	3	9

^a^ Fully vaccinated is defined as having received the complete immunization series according to the vaccine manufacturer.

^b^ Self-reported symptoms.

^c^ Cases may exhibit multiple symptoms.

^d^ Includes passengers with flights originating in the Republic of Korea (*n* = 3) as well as passengers with layovers in the Republic of Korea (*n* = 13) whose flights originated in the United States of America (*n* = 9), Canada (*n* = 3) or the Netherlands (*n* = 1).

Sequencing results were reported promptly to the originating province and the MoH, which shared them publicly via Internet news outlets. All sequences were also uploaded to GISAID within 2 weeks; GISAID accession numbers are listed in **Supplementary Fig. 2**.

## Discussion

Viet Nam’s practice of universal testing and mandatory quarantine for international visitors, combined with preferential selection of imported cases for whole genome sequencing, enabled the early detection of imported Omicron cases. Prompt identification and isolation of these cases gave the government additional time to communicate with the public and prepare for eventual community transmission. This supports previous studies demonstrating that public health and social measures such as systematic targeted testing programmes remain important, even among highly vaccinated populations such as travellers. (*4*) Multiple flight origins further highlight the need for cooperation between countries to detect and respond to emerging variants. Testing and sequencing strategies should be revisited and updated as outbreak situations continue to evolve.

Government policies prioritizing whole-genome sequencing may improve the speed of variant detection, and collecting epidemiological data provides important context for sequenced cases. For example, the observation that most cases in this series were fully vaccinated supports previous data demonstrating that vaccination does not prevent transmission of the Omicron variant. (*5*) Similarly, the lack of hospitalization or death supports data that Omicron causes severe illness less frequently than Delta. (*6*) The integration of laboratory and epidemiological data is critical in ensuring the most useful information is available as quickly as possible, although the low proportion of specimens sequenced compared to total cases limits interpretation.

As of 14 August 2022, Viet Nam reached 84.1% population coverage with two vaccine doses, allowing the country to slowly re-open to international travel and commerce. (*7*) Genomic surveillance for SARS-CoV-2 has become the international standard of surveillance. (*8*) In addition to contributing to the understanding of clinical and epidemiological trends of COVID-19, genomic surveillance provides critical data necessary for rapidly developing newer, more effective vaccines against SARS-CoV-2.

Early investment in infrastructure for genomic sequencing made it possible for authorities in central Viet Nam to respond quickly to the detection of a newly imported SARS-CoV-2 variant of concern. However, implementing new programmes always comes with challenges. For example, current limitations include overrepresentation of specimens from certain provinces and underrepresentation from others, as well as resource constraints at the local level for specimen collection and transfer. Despite these issues, the data described here provide valuable evidence for further investment to overcome current limitations and scale up towards the greater goal of developing a National Sequencing Network in Viet Nam.
